# Efforts to Improve the Seasonal Influenza Vaccine

**DOI:** 10.3390/vaccines6020019

**Published:** 2018-03-30

**Authors:** Alfred T. Harding, Nicholas S. Heaton

**Affiliations:** Department of Molecular Genetics and Microbiology, Duke University, Durham, NC 27710, USA; alfred.harding@duke.edu

**Keywords:** influenza, seasonal vaccine, egg-adaptation, vaccine efficacy

## Abstract

Influenza viruses infect approximately 20% of the global population annually, resulting in hundreds of thousands of deaths. While there are Food and Drug Administration (FDA) approved antiviral drugs for combating the disease, vaccination remains the best strategy for preventing infection. Due to the rapid mutation rate of influenza viruses, vaccine formulations need to be updated every year to provide adequate protection. In recent years, a great amount of effort has been focused on the development of a universal vaccine capable of eliciting broadly protective immunity. While universal influenza vaccines clearly have the best potential to provide long-lasting protection against influenza viruses, the timeline for their development, as well as the true universality of protection they afford, remains uncertain. In an attempt to reduce influenza disease burden while universal vaccines are developed and tested, many groups are working on a variety of strategies to improve the efficacy of the standard seasonal vaccine. This review will highlight the different techniques and technologies that have been, or are being, developed to improve the seasonal vaccination efforts against influenza viruses.

## 1. Introduction

The family *Orthomyxoviridae* is composed of seven genera of negative sense, segmented, single-stranded RNA viruses [1]. Influenza A and B viruses (IAV and IBV respectively) are responsible for the vast majority of the estimated 3 to 5 million cases of influenza-mediated severe illness and 290,000–650,000 deaths annually [2]. To date, vaccination remains the best strategy for preventing the spread of IAV and IBV [3]. The influenza vaccine is currently administered in a “trivalent” or “quadrivalent” format, wherein the vaccine is a cocktail of two IAV strains and either one or two IBV strains [4]. Due to rapid viral evolution, unlike many other vaccines, the influenza vaccine formulation requires updates each flu season. Data from the WHO Global Influenza Surveillance and Response System ensures the strains in the vaccine match circulating viruses; hence, the term “seasonal vaccine” [5]. This need for continual adjustment of the vaccine formula is a result of influenza viruses’ ability to accumulate mutations over time, a process termed “antigenic drift” [6]. Influenza viruses, like many RNA viruses, have a much higher mutation rate than organisms with DNA genomes due to the lower fidelity of RNA polymerases [7]. Intrinsic viral mutation combined with immune selective pressures result in the fixation of viral variants that are antigenically distinct from their predecessors. These “drifted” viruses are frequently capable of escaping the immune response elicited by the previous vaccination or infection, leading to the requirement for constant monitoring and testing of isolated strains to ensure the current vaccine is a match to circulating viruses. Vaccine mediated protection against influenza viruses is further complicated by the fact that the segmented viral genome permits two different virus strains to reassort genetic material with one another upon coinfection, potentially leading to “antigenic shift” [8]. Antigenic shift enables the creation of antigenically novel viral strains capable of causing pandemic outbreaks. Thus, seasonal influenza vaccine production must be flexible enough to deal with the annual acquisition of mutations in circulating strains and to rapidly respond to pandemic outbreaks as occurred with a reassortant H1N1 strain in 2009 [9].

Rapid influenza virus evolution leads to the yearly requirement for massive vaccine manufacturing infrastructure capable of generating hundreds of millions doses. Currently, the majority of influenza virus vaccines are manufactured using embryonated chicken eggs [10]. This manufacturing strategy, which has been used for ~50 years, begins with the identification of the predicted circulating strains. Quadrivalent vaccines today are composed of the two-major circulating IAV strains, H1N1 and H3N2 viruses, and the two major lineages of IBV viruses, Yamagata and Victoria [11,12,13]. Once a strain is selected, the genetic segments encoding the two major glycoproteins, hemagglutinin (HA) and neuraminidase (NA), of the circulating influenza viruses are reassorted into an egg-adapted background virus containing the remaining 6 segments [14]. Once these reassortant viruses are generated, they are grown in embryonated chicken eggs and screened to isolate “candidate vaccine viruses” or CVVs, that grow to high-titers in eggs and remain antigenically similar to the circulating strains [5]. The CDC, or another center affiliated with the WHO Global Influenza Surveillance and Response System, then delivers the approved CVVs to private manufacturers [10,15]. These manufacturers then mass-produce the viruses in embryonated chicken eggs and subsequently partially purify the reassortant viruses. These purified viral particles are then inactivated and standardized based on HA content [16]. The HA protein is primarily responsible for inducing neutralizing antibodies against influenza viruses, and as a result is the focus of most vaccines [17,18].

This manufacturing technique has remained relatively unchanged across decades for a number of reasons. Firstly, vast infrastructure for producing egg-based influenza vaccines currently exists and is required to meet the annual need of new seasonal vaccines for the global population [19]. It is estimated that the current egg-based manufacturing industry is capable of producing 1.5 billion doses annually, a number other vaccine manufacturing techniques have difficulty matching [20]. Secondly, due to its extraordinary manufacturing capacity and robust viral growth, egg-based influenza vaccines are also among the cheapest vaccines available [21,22]. Low cost vaccines allow protection from influenza disease in both developed and developing nations.

**Figure 1:**
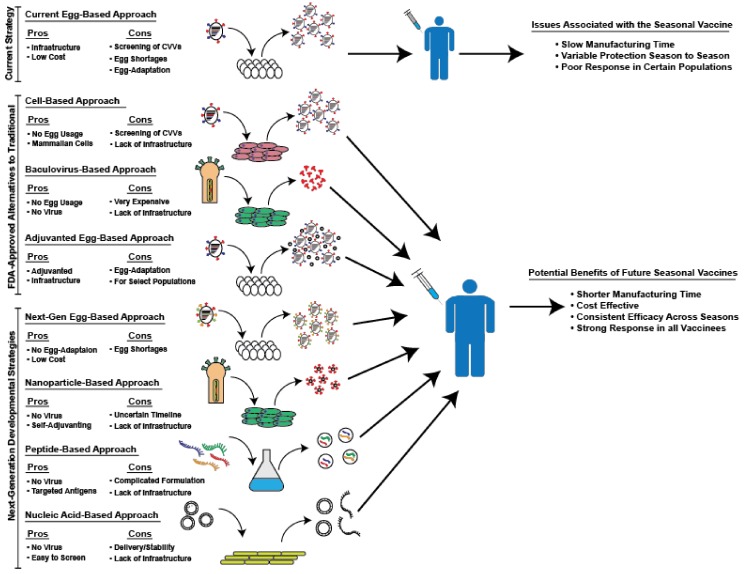
Summary of alternative approaches to traditional, egg-grown seasonal influenza vaccines that are either currently in use or in development. A depiction of the various alternative approaches to avoid the problems associated with the current seasonal influenza vaccines and a list of their respective pros and cons.

Although egg-based manufacturing confers several benefits, serious drawbacks remain. First of all, reassortant viruses must be adapted in eggs to produce high-yield candidate vaccine viruses. This process, combined with the need to screen the antigenicity of isolated strains, drastically increases the production time of influenza vaccines for the upcoming season [22,23,24]. Increased production time reduces the flexibility of the manufacturing process, necessitating the start of production long before the start of the season. This timeline reduces the ability of public health officials to adapt to sudden changes in circulating strains. This process of egg-adaptation is both slow and, at times, ineffective. Despite efforts to adapt reassortant viruses to culture in eggs, some strains, especially H3N2 viruses, continue to grow poorly in eggs [25]. This inability to adapt strains can result in significant delays in vaccine production due to the lower yield of these strains and, in some severe cases, may necessitate the removal of a predicted strain due to its inability to be grown to sufficient levels [25,26]. This problem occurred during the 2003–2004 season, when the predicted A/Fujian/411/2002 strain was unable to be grown successfully in chicken eggs and was subsequently replaced by the prior year’s H3N2 strain [27]. Greater than 82% of the isolates examined by the CDC from that year antigenically matched the A/Fujian/411/2002 strain, resulting in epidemic levels of influenza morbidity and mortality [28]. Additionally, it was recently shown that egg-adaptation can negatively impact vaccine effectiveness [25,29,30]. The viral hemagglutinin (HA), the primary antigenic target of neutralizing antibodies, binds to sialic acid on the surface of host cells to facilitate influenza virus entry [1]. The sialic acids on human cells in the upper respiratory tract exhibit an α-2,6 linkage, whereas avian cells exhibit an α-2,3-linkage, resulting in a different conformation for recognition by the HA protein [31,32]. Poor receptor binding is a primary reason that many human influenza viruses exhibit poor growth in eggs, inducing a selective pressure on these viruses to adapt their HA proteins. Unfortunately, the binding region of the HA protein is in the globular head domain, which contains the major antigenic sites which are targeted by neutralizing antibodies [1]. Thus, many of the mutations influenza viruses accumulate during egg-adaptation result in altered antigenicity. This potentially changed antigenicity is usually controlled for during the generation of CVVs by continually testing the antigenicity of isolated strains. However, due to the complex nature of influenza immune responses in individuals with multiple exposures to different strains, it can be difficult to accurately predict the antigenicity of a given vaccine strain [33]. Furthermore, CVVs are not closely monitored during egg-based manufacturing and unstable vaccine strains can result in the production of mutated vaccine viruses that no longer represent circulating strains [25,29]. These instances of egg-adaptation, notable in recent years, have contributed to poor vaccine efficacy as a result of antigenic mismatch of adapted viruses to circulating viruses [34]. For the current 2017–2018 influenza season, it was demonstrated that a single amino acid mutation acquired during egg-adaptation, which changed the glycosylation of the HA protein, is likely responsible for the estimated 25% vaccine effectiveness against the circulating H3N2 virus in adults in the U.S. [29,35]. This review will focus on novel strategies that have been developed to address the issues associated with egg-based production in an effort to produce a more effective seasonal influenza vaccine. These strategies and their respective potential benefits and drawbacks, are briefly schematized in Figure 1.

## 2. Currently Available, FDA-Approved Alternatives to Traditional Egg-Based Vaccines

In 2012, the FDA announced its approval of Flucelvax^®^, the first approved non-egg produced vaccine alternative in the US [36]. Flucelvax^®^ is a cell-based influenza vaccine manufacturing platform, developed by Novartis’s influenza vaccine group (now owned by Seqirus), wherein influenza viruses are grown in tissue culture systems using Madin-Darby Canine Kidney (MDCK) cells [37]. Similar to egg-based production, reassortant viruses are generated using the HA and NA of clinically relevant strains in a standardized genetic backbone. The four selected CVVs are then amplified, the viruses are purified, and finally inactivated for use in vaccines [38]. This strategy, while similar to the egg-based manufacturing process, has several advantages over egg growth. Importantly, the utilization of cells reduces the potential constraints of egg-shortages. Egg-based manufacturing techniques are reliant on an enormous supply of chicken eggs, sometimes slowing production time based on their availability. In contrast, cell-based manufacturing offers a more flexible production timeline since virus amplification is dependent on the capacity of bioreactors [38]. Another advantage is based on data showing that the glycosylation of the HA protein can drastically impact immunogenicity [29]. Previous work demonstrated that virus grown in different cell types, for example, avian (egg) compared to mammalian, may exhibit drastically different glycosylation profiles [39,40]. While not yet formally shown, many groups are investigating the theory that utilizing mammalian cells as a substrate, rather than avian cells, will yield more antigenically matched HAs for vaccines. Furthermore, because this strategy uses cells and not eggs, there is no risk of egg-allergies negatively impacting patients who have previously experienced anaphylaxis. An additional, and perhaps the most appealing, advantage of vaccine production in mammalian cells is the reduction of the egg selective pressures driving HA mutations. Originally, the same CVVs used in egg-based manufacturing were also utilized for the production of Flucelvax^®^. This meant in years where egg-adaptation was an issue, the cell-based vaccine was also impacted [34]. In 2016, however, the FDA approved the use of cell-based CVVs and has since mandated that all Flucelvax^®^ doses be made exclusively using cell-based CVVs to ensure its insulation from egg-adaptation [34]. In fact, Scott Gottlieb, the Commissioner of the Food and Drug Administration, recently stated that “about 20 percent improved efficacy for the cell-based vaccine relative to the egg-based vaccines” has been estimated by the FDA for the 2017–2018 season [41].

While this platform offers a number of advantages, drawbacks remain. Concerns around adaptation remain when using MDCK cells. It has recently been shown that influenza viruses can develop mutations in the HA and NA proteins after serial passaging in cell culture [42]. While it needs to be determined whether these mutations happen during vaccine manufacturing, or impact vaccine effectiveness, this finding highlights an important area of research as this strategy becomes more prevalent. Additionally, unlike the egg-based vaccines, the global-scale infrastructure for manufacturing the necessary amount of cell-based influenza vaccines does not currently exist. According to David Minella, a communications manager for Seqirus, approximately “21.5 million doses of [Flucelvax] were provided to the U.S.” for the 2017–2018 influenza season (personal communication). This number, roughly equivalent to Seqirus’ current annual manufacturing capacity, represents only 18% of the estimated quadrivalent vaccine supply that was available in the U.S. for the 2017–2018 influenza season [43]. Also, according to the CDC vaccine pricelist Flucelvax^®^ can cost up to 40% more than an egg-based vaccine. Removing the requirement and risks of egg adaption demonstrates the appeal of a cell-based design, yet it is clear this system cannot currently replace the entirety of egg-based manufacturing at a low cost without significant investments in infrastructure.

Another method to avoid egg-based manufacturing eliminates the reliance on influenza virus replication/production entirely. Only a year after Flucelvax^®^ was approved, the FDA also announced the approval of Flublok^®^, made by Protein Sciences Corporation [44]. Unlike both egg-based and cell-based manufacturing, Flublok^®^ utilizes baculovirus-expression systems to purify recombinant HA protein [45,46]. Manufacturers clone a desired HA gene into a baculovirus transfer vector, using RT-PCR from viral RNA, or synthesize it using a known sequence [45]. Once generated, manufacturers transfect the cloned HA gene and linearized baculovirus genomic DNA into insect cells. This process allows recombinant baculoviruses to form and subsequently be used as stock viruses. Stock viruses infect insect cells in bioreactors to produce recombinant HA protein that is purified and used for vaccination. This manufacturing strategy is much quicker since there is no longer a need to rescue influenza viruses and select for high yield variants. This efficiency creates a much more flexible platform, allowing public health officials to potentially adapt to sudden changes in circulating influenza strains. This strategy is also immune to the issues of egg-adaptation, as it produces an HA with an exact amino acid match to the circulating virus [46]. Furthermore, this strategy allows for the production of vaccines with higher HA concentrations. Optimized purification techniques and the sole expression of HA allow manufacturers to produce exceptionally pure samples of antigen that can be concentrated to much higher levels, while simultaneously maintaining low levels of contaminant [47]. Lastly, similar to the cell-based strategy, this system does not utilize eggs, insulating it from the limitations of egg-shortages and the potential issue of severe allergic responses to egg proteins in some patients. Advantages aside, this strategy is not immune to drawbacks, especially with regard to cost. According to the CDC, Flublok^®^ can cost more than twice as much as an egg-based vaccine, causing many patients to opt for either the egg or cell-based vaccines [48]. Flublok^®^ also has the shortest shelf-life out of the three vaccine strategies. It is not recommended to be stocked for longer than nine months, requiring a restocking of the vaccine regardless of whether the formulation is changed in a given year [44]. Furthermore, similar to the cell-based strategy, the infrastructure to manufacture enough doses for an entire season does not yet exist. Without a low-cost production scheme and extended shelf-life, it is unlikely that Flublok^®^ could completely replace egg-based manufacturing.

The final FDA-approved alternative to traditional egg-based production methods works to simply improve the protection afforded by the standard vaccine. Instead of relying on a different production method, the approach is to incorporate an adjuvant to increase the magnitude of the immune response against the vaccine antigens. FLUAD^®^, which was originally developed by Novartis’s influenza group (now owned by Seqirus), was approved in the U.S. for use by patients 65 and older in 2015, with distribution starting during the 2016–2017 influenza season [49]. The FLUAD^®^ vaccine has actually been approved for use in other countries since the late 1990’s, with over 85 million doses distributed worldwide [50,51]. While this vaccine platform still relies on egg-based manufacturing, it incorporates the use of an emulsion-based adjuvant, MF59 [52]. This emulsion is comprised primarily of squalene, an organic compound commonly utilized in the human body to synthesize cholesterol and other steroids [52]. MF59 enhances immune responses by inducing the recruitment of immune cells to the injection site, allowing an enhanced uptake of and subsequent response to the antigen [51]. While this vaccine is currently only approved in patients 65 and older, recent studies have begun to demonstrate its ability to induce robust immune responses in children [53]. Studies have also shown the addition of MF59 was capable of inducing cross-reactive antibodies able to neutralize strains not included in the vaccine [53,54]. FLUAD^®^, while capable of inducing stronger responses than un-adjuvanted vaccines, is still based on growing influenza viruses in eggs, making it susceptible to viral adaptations during growth in eggs. The only additional issue with employing the widespread use of adjuvants like MF59 is the commonly mentioned mild side-effect of enhanced pain at the injection site, likely due to the increased influx of immune cells [49].

## 3. Next-Generation Seasonal Influenza Vaccines Currently in Development

**Figure 2:**
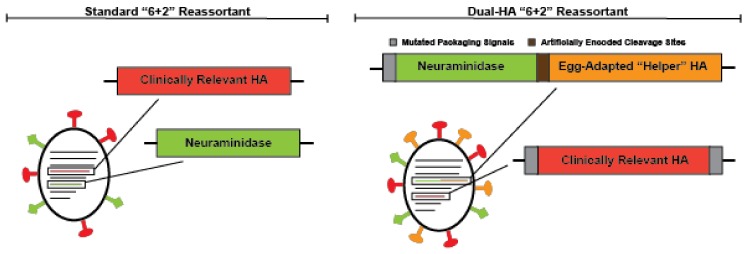
A schematic of the standard “6 + 2” reassortant virus and the dual-hemagglutinin (HA) design.

Due to the challenges associated with the current FDA approved alternative (non-egg produced) influenza vaccines described above, the use of these vaccines has remained relatively limited when compared to standard egg-grown vaccines. Thus, alternative strategies to improve the seasonal influenza vaccine are continuing to be developed. While many groups have simply avoided egg-based growth strategies to overcome the limitations of the traditional influenza vaccine, our group has focused on engineering recombinant viruses with desired characteristics for vaccine production. We recently reported a genomic organization for the influenza virus vaccine backbone that can be grown in chicken eggs, yet avoids egg-adaptive mutations [55]. To accomplish this goal, we expressed both an HA and NA protein from a single genomic segment, which normally only encodes the HA protein, and then expressed a second HA from the segment once occupied by NA (Figure 2). This scheme allows us to express an egg-adapted “helper” HA and a clinically relevant HA on the same virion. The “helper” HA eliminates the selective pressure on the clinically relevant HA, which would likely be unstable during growth in eggs. We were able to show that an H3N2 strain, which normally grows extremely poorly in eggs and subsequently acquires a number of well characterized HA mutations [30,56,57], when placed in our dual-HA background could grow to high titers without a single adaptive mutation in the H3 HA [55]. Pairing egg-adapted helper HAs dramatically enhances the growth of HA proteins known to grow poorly in chicken eggs. In theory, eliminating the need to adapt and screen reassortants for high-yield CVVs could significantly reduce vaccine production timelines. Without egg-adaptation, dual-HA vaccines may elicit better matched immune responses against circulating strains. In contrast to all other alternative strategies, our design is compatible with current manufacturing processes, and thus, could utilize the established egg-based manufacturing infrastructure [55]. Remaining reliant on eggs means this platform is still susceptible to egg shortages and inappropriate for people with a history of severe egg allergies. However, this system would immediately allow large-scale manufacturing of the vaccine at the same prices currently available for standard egg-based formulations.

Another alternative strategy currently under development uses nanoparticles. Novavax Inc. had promising preclinical trials for its nanoparticle-based influenza vaccine platform, NanoFlu^®^ and will soon be moving into phase I studies [58]. Similar to the baculovirus system, nanoparticle-based approaches depend on the expression of the desired antigen, typically HA, in a cell line [58,59]. Once expressed, the HA is purified and assembled into a nanoparticle for use in the vaccine. Due to the similarities to the baculovirus based production methods, nanoparticle methods retain many of the same advantages, including the ability to generate a large quantity of exact sequence HA, insulating this strategy from egg-adaptation/mutation [60]. Unlike the currently approved Flublok^®^, this strategy uses a full particle as opposed to HA protein in solution. It has been demonstrated that presenting antigens in this particulate form often yields boosted immune responses, acting as a self-adjuvant [61,62]. Additionally, nanoparticle-based strategies show promise as universal vaccines, with several eliciting a broader, more universal anti-influenza immune response [58,59]. Nanoparticles’ ability to elicit broadly reactive anti-viral antibodies could allow a given nanoparticle seasonal vaccine to protect for a longer period of time compared to a standard seasonal influenza vaccine. While this strategy demonstrates promise, there is currently a lack of infrastructure for the manufacturing, as with the other non-egg grown vaccines, needed to fulfill the necessary doses for a season. Additionally, there is a less established precedent for nanoparticle-based vaccines, which may increase the timeline for regulatory approval.

Peptide-based vaccines present another opportunity for improvement over current production methods. This platform relies on the synthesis of specific epitopes from influenza proteins (often HA, M1/M2 and NP) recognized by B- and T-cells [63,64,65]. Once synthesized, peptides are purified and loaded into either liposomes or virosomes, which serve as both an adjuvant and a delivery mechanism for the antigens [64,66]. Liposomes and virosomes have both been demonstrated to effectively target cells of interest and deliver antigen. Currently, many groups are working to evaluate what delivery strategy might better suit vaccination needs [67,68]. Both liposome and virosome formulations of these vaccines have undergone pre-clinical trials and demonstrated the ability to elicit protection from subsequent influenza virus challenge [63]. Peptide vaccines are insulated from the issue of egg-adaptation by relying on peptide synthesis, and avoid other egg-based risks such as egg-availability or egg-allergens. Peptide delivery formulations are also capable of serving as an adjuvant in addition to delivering the antigens [68,69]. Lastly, similar to the nanoparticle vaccine, these strategies have shown a potentially more universal protection by targeting conserved epitopes of the desired antigen [70]. However, this strategy, requires further development before its widespread use. The formulation of these vaccines is very complicated, as the it includes both the antigenic peptides and the composition of the liposome/virosome itself [63,67]. Optimization of these components can take significant time and, as with other experimental approaches, the true costs are unknown and the infrastructure for manufacturing these vaccines is currently limited.

Another promising approach in development are nucleic acid-based vaccines. Unlike any of the previously mentioned strategies, nucleic acid-based vaccines do not rely on the production of proteins but instead recombinant DNA or RNA molecules [71,72,73]. In either case, the sequence of the desired antigen is cloned into an expression plasmid and propagated in bacterial cells, typically *E. coli*. For RNA vaccines, a transcription step follows the initial replication of the DNA template, which is subsequently degraded using DNAses. The RNA or DNA is then purified and often administered via injection. Host cells take up the RNA or DNA and begin expressing the desired antigen. These approaches have been used in a variety of preclinical and phase 1 clinical trials, typically expressing at least the HA protein of the desired virus and show a great deal of promise for future clinical trials and subsequent development [74,75,76,77,78,79]. While there are some major differences between DNA and RNA-based vaccines, they share many of the same advantages. Similar to other expression-based platforms, DNA and RNA vaccines are immune to the potential pitfall of protein adaptation during manufacturing processes. Synthesis of the nucleotide sequence guarantees that the expressed antigen is identical to its circulating target. Nucleic acid-based vaccines are also able to be rapidly manufactured [71]. These strategies only require the manufacturing of nucleic acid, not protein, effectively removing a step of the manufacturing process. Furthermore, when considering the ease of amplifying and purifying nucleic acid, it becomes clear that screening and expressing new antigens and or adjuvant-antigen combinations would become much faster and more efficient than in any of the other strategies described [71]. Lastly, delivery of these vaccines can be tailored to specific cell types by utilizing an assortment of delivery vectors [80]. Delivery can be especially simple for RNA-based vaccines, as it has been shown that cells are capable of spontaneously taking in naked mRNA [81,82]. Despite ease of production and delivery, many challenges for nucleic acid-based vaccines remain. This class of vaccines have not previously been approved for use in humans, although they have been approved for veterinary use [83]. Furthermore, nucleic acid introduction into the cell can activate a number of innate immune signaling pathways [84]. While nucleic acid vaccines have shown promise in preclinical trials, it was shown that activation of the innate response by these vaccines can drastically reduce their efficacy [71]. Strategies to control innate immune recognition of these molecules, such as the incorporation of non-immunogenic pseudouridine bases into mRNAs [85], will be critical to ensure that these vaccines express sufficient antigen to induce protective immunity.

## 4. Conclusions

Despite the fluctuating efficacy of the seasonal influenza vaccine from year to year, it remains the best strategy for combating infection. Experimental universal influenza vaccines are intended to broadly protect against many (if not all) influenza virus strains regardless of antigenic mutation in the HA head domain (reviewed in [86,87,88]). As with the development of any new vaccine, the timeline for their widespread use in humans, as well as their true efficacy against divergent viral strains is uncertain. Thus, as a short-term measure, efforts to improve the efficacy of the seasonal vaccine as well as the development of other anti-viral therapeutics are still needed. The development and application of new approaches to improve on the current technologies, along with the development of completely new vaccines, makes this an exciting time to be part of the influenza virus research community. Current efforts and further optimization of many complementary strategies for influenza vaccine development are critical to our ability to reduce and even prevent the epidemic and pandemic outbreaks of the future.
